# Multifolding Vertical-Flow Electrochemical Paper-Based Devices with Tunable Dual Preconcentration for Enhanced Multiplexed Assays of Heavy Metals

**DOI:** 10.1021/acs.analchem.4c06982

**Published:** 2025-01-09

**Authors:** Dionysios Soulis, Electra Mermiga, Varvara Pagkali, Maria Trachioti, Christos Kokkinos, Mamas Prodromidis, Anastasios Economou

**Affiliations:** † Laboratory of Analytical Chemistry, Department of Chemistry, University of Athens, Athens 157 71, Greece; ‡ Laboratory of Analytical Chemistry, Department of Chemistry, University of Ioannina, Ioannina 45110, Greece

## Abstract

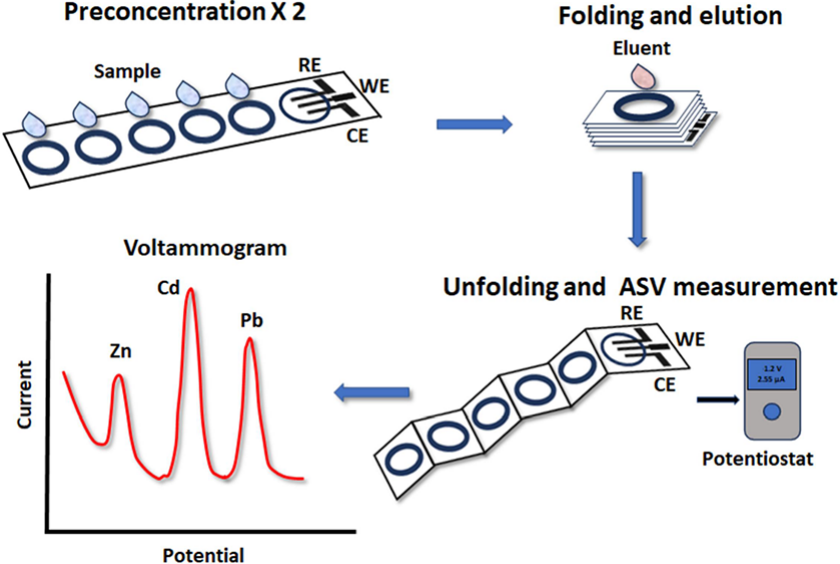

This work describes fully integrated multifolding electrochemical paper-based devices (ePADs) for enhanced multiplexed voltammetric determination of heavy metals (Zn(II), Cd(II), and Pb(II)) using tunable passive preconcentration. The paper devices integrate five circular sample preconcentration layers and a 3-electrode electrochemical cell. The hydrophobic barriers of the devices are drawn by pen-plotting with hydrophobic ink, while the electrodes are deposited by screen-printing. The devices exploit the wicking ability of cellulose paper to perform passive preconcentration of the target analytes, resulting in a ∼6-fold signal enhancement. For this purpose, drops of the sample are placed at the five sample pads of the preconcentration layers, the device is folded, and the target metals are eluted in a vertical-flow mode to the electrochemical cell, where they are measured directly by anodic stripping voltammetry (ASV). The working electrode of the ePADs is bulk-modified with bismuth citrate; during the ASV measurements, the bismuth precursor is converted to nanodomains of metallic bismuth at the surface of the working electrode. By combining the triplex signal amplification through passive preconcentration, electrochemical preconcentration, and judicious working electrode modification with *in situ* generated bismuth nanoparticles, ultrasensitive and multiplexed heavy metal assays can be achieved. Due to their high degree of integration, low cost, easy and fast fabrication, and sensitivity, the multifolding ePADs are particularly suitable for on-site heavy metals' monitoring applications.

## Introduction

Over the past few years, environmental contamination has become an issue of increasingly significant environmental and health concerns worldwide.^1−7^ Therefore, strict legislation has been established by national and international authorities specifying the maximum limits of several heavy metals in various matrices with particular emphasis on environmental and food samples.^7−9^

Different variants of atomic spectroscopy are commonly used for trace determination of heavy metals in most relevant samples, but these are not suitable for on-site rapid assays due to the expensive instrumentation involved and the high purchase/running costs in addition to the requirements for laboratory facilities, laborious and time-consuming sample preparation, and trained personnel.^10,11^ In contrast, electrochemical stripping analysis enables fast, ultrasensitive field assays of heavy metals thanks to its high sensitivity and miniaturized instrumentation.^12−14^

Several electrochemical paper-based analytical devices (ePADs) have been developed for stripping analysis of heavy metals^15−34^ by capitalizing on the manyfold advantages of cellulose paper as an analytical platform.^35−38^ Although the aforementioned devices are useful in their own right, several challenges still remain to be tackled.^39^

A major challenge is the issue of sensitivity. The first route toward increasing the sensitivity in stripping analysis of heavy metals with ePADs is to increase the preconcentration efficiency. The high sensitivity of stripping analysis is mainly due to the preconcentration step applied prior to the voltammetric detection step under conditions of convection.^40−42^ This process is much less efficient in paper devices because the cellulose fibers disrupt the diffusion process;^39^ thus, the limits of detection (LODs) for heavy metals achieved with paper-based devices are typically at least 1 order of magnitude higher than the LODs attainable using conventional cell arrangements. In order to improve the sensitivity, different strategies have been developed for coupling preconcentration techniques with paper-based devices.^43^ In the context of stripping analysis of heavy metals, the most straightforward solution is to promote the sample flow within the network of cellulose fibers using microfluidic arrangements.^26,33^ Another methodology is to amplify the preconcentration efficiency using heating of the sample, but this approach requires additional external equipment.^20^ The most promising methods for sample preconcentration are those that are exclusively passive and exploit the wicking ability and conformability of paper.^18,32^

A second path to increasing the sensitivity is to amplify the electrochemical signal through the use of modified electrodes. Since the use of mercury is strictly regulated,^44^ electrodes made of, or modified with, bismuth have been shown to provide exceptional sensitivity^45−47^ and, indeed, some applications of ePADs with bismuth-based sensors exist in the literature.^16,19,27,28^ However, the existing ePADs are based on *in situ* or *ex situ* electroplating of the working electrode with bismuth, which is a challenging task because it requires the use of a Bi(III) solution and additional steps, while, in most cases, LODs still cannot comply with the current legislative limits for heavy metals.

A second challenge is to achieve multiplexed detection of more than one heavy metal in a single run which is highly desirable on the grounds of cost, speed, and information content per analysis.^48^ However, applications with paper-based devices are limited to one or two target metals with multiplexed applications of more than 2 metals being very scarce.^29^

The final challenge is related to device fabrication. The majority of the existing relevant reports employ wax printing to form hydrophobic barriers, but wax printers are not commercially available anymore, while wax-printed PADs require post-fabrication treatment (to allow melting of the wax and penetration in the paper matrix).^49,50^ A more serious issue pertaining to device fabrication is the degree of integration since it is highly desirable to produce devices in which the electrochemical cell is entirely integrated into the paper device for the sake of cost, simplicity, and speed of fabrication, convenience of the assay, and practicality (i.e., no need for manual alignment and attachment of the two modules). However, the vast majority of existing ePADs for stripping analysis of heavy metals are not fully integrated and employ modular construction in which the electrochemical cell is fabricated separately on a different (usually plastic) substrate and physically attached to the paper-based fluidic or disk.^15−17,19−21,23,27−29,33,34^ Only a handful of truly integrated ePADs exist, but these have not been applied to multielement determinations and the LODs are relatively high.^22,24,26,31^

This work describes a multifolding ePAD that was designed and implemented for the voltammetric determination of trace metals in such a way as to successfully address the aforementioned limitations and challenges associated with conventional ePADs. The goal was to manufacture integrated ePADs for multiplexed heavy metal detection that could achieve sub-μg L^–1^ LODs by using sustainable and rapid fabrication methods. The devices exploit an *in situ* tunable dual passive preconcentration approach of the target analytes, followed by a self-driven elution step. The hydrophobic barriers of the ePAD, instead of wax printing, were formed by pen-plotting with a commercial hydrophobic marker pen,^51^ while screen-printing was used for the deposition of the electrodes of the electrochemical cell.^52^ The combination of pen-plotting and screen-printing enables the fabrication of entirely integrated devices in a convenient, low-cost, and rapid manner. In order to further amplify the signal, to achieve simultaneous detection of Zn(II), Cd(II), and Pb(II), and to simplify the analytical protocol, the screen-printed working electrode of the devices was bulk-modified with bismuth citrate as a bismuth precursor.^53^

## Experimental Section

### Chemicals and Reagents

All of the chemicals were of analytical grade and purchased from Merck (Germany) or Sigma-Aldrich (St. Louis, USA). Doubly distilled water was used throughout. Stock solutions containing 10 and 100 mg L^–1^ of different metals (Bi(III), Mn(II), Mg(II), Fe(III), Ni(II), Cd(II), Pb(II), Sn(II), Zn(II), Cu(II), and Sb(III)) were prepared from 1000 mg L^–1^ standard solutions after appropriate dilution with water. A 2.0 mol L^–1^ acetate buffer (pH 4.5) was prepared from sodium acetate and hydrochloric acid.

Macherey-Nagel chromatography paper MN 261 served as the substrate and BIC Marking Pro ultraresistant permanent marker (1.1 mm tip) was used to form the hydrophobic barriers. Conductive graphite ink (Loctite EDAG 423SS, Henkel, Belgium) was used to screen-print the electrodes.

### Instrumentation

The fluidic patterns were drawn using the open-access software Inkscape version 1.0.1 (Inkscape Project, https://inkscape.org/about/). The AxiDraw extension for Inkscape was used for controlling an AxiDraw desktop *x*–*y* plotter (Evil Mad Science LLC, Sunnyvale, CA) connected to a PC via a USB interface.

A semiautomatic screen printer (E2, EKRA), polyester screens (77/195-48 PW, SEFAR PET 1500), and a 75-durometer polyurethane squeegee were used for screen-printing of the electrodes.

Scanning electron microscopy (SEM) images were taken with a Phenom Pharos G2 (Thermo Fisher Scientific) desktop instrument on chromium-sputtered samples prepared with a Quorum Q150T ES plus device.

For the electrochemical measurements, a Palmsens potentiostat controlled by PSTrace 5.9 software (Palmsens, Houten, the Netherlands) was used. All electrochemical data evaluation was performed with the PSTrace 5.9 utilities.

### Multifolding ePAD Fabrication

**Figure 1:**
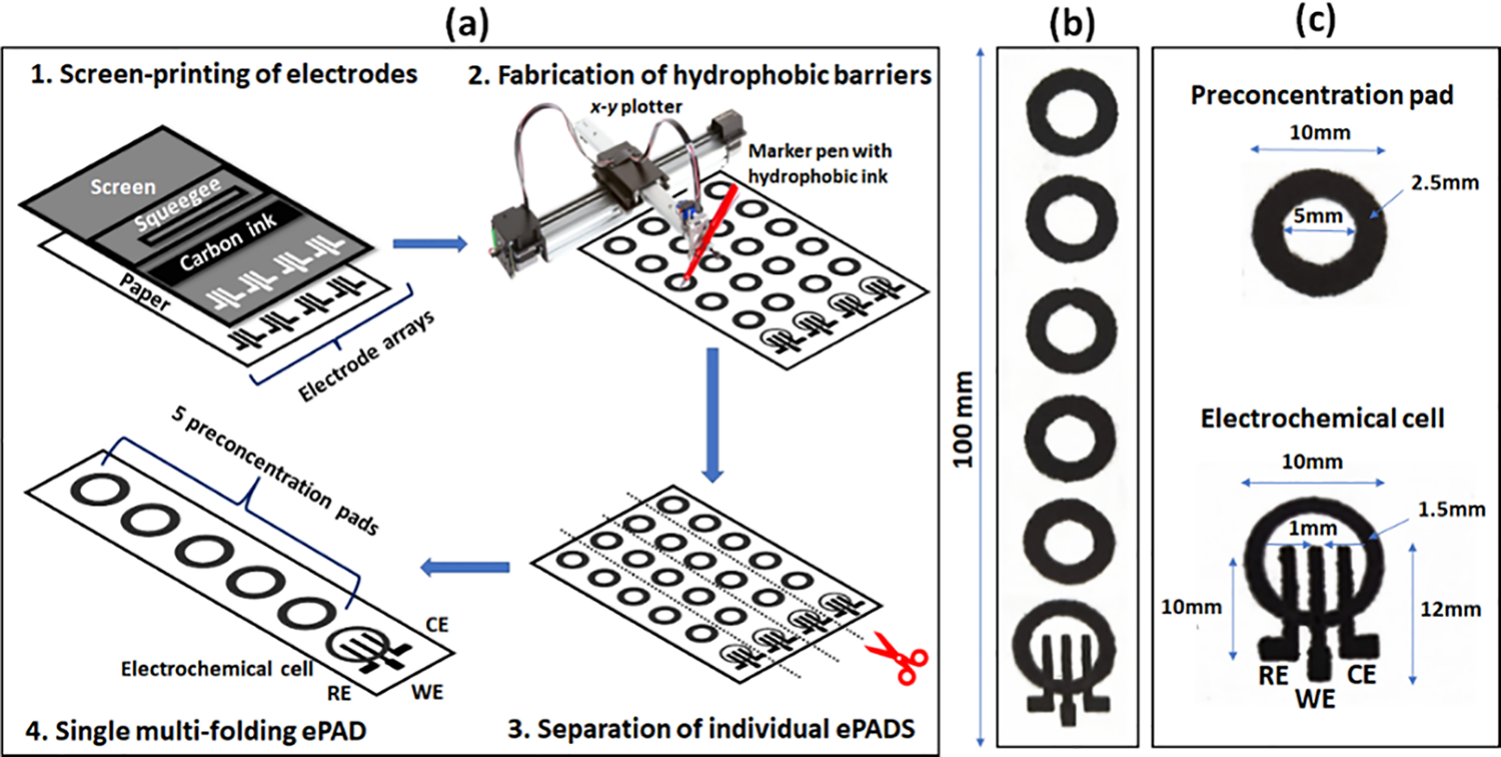
(a) Fabrication of the multifolding ePADS, (b) photo of a single multifolding ePAD, and (c) nominal dimensions of the ePAD components (WE is the working electrode; RE is the reference electrode; and CE is the counter electrode).

The fabrication of the ePADs proceeded in three steps (Figure 1a).

a) Deposition of the three-electrode arrays. The three electrodes were directly printed on the paper substrate. Initially, a layer of graphite ink was deposited to pattern the three electrodes. For the experiments involving a bismuth citrate-modified working electrode, a second layer of graphite ink containing 6% (*w/w*) of bismuth citrate was screen-printed on top of the working electrode. After each printing step, the ink was cured at 90 °C for 5 min using an infrared curing system (LittleRed-X2, VASTEX).
b) Formation of the sample pads and the electrochemical cell. The paper with the arrays of the three-electrode was positioned onto a flat glass surface and aligned with the aid of preset alignment marks drawn on the paper sheets and the glass surface. The marker pen was inserted into the holder of the plotter, and the sample pads and the borders of the electrochemical cells were drawn on the paper using a plotting speed of 0.76 cm s^–1^ and left at room temperature for 5 min to allow the solvent to evaporate.
c) Separation of individual ePADs. Finally, the paper was cut using scissors to obtain the individual multifolding ePADs. A photo and the nominal dimensions of the components of the ePADs are shown in Figure 1b,c.

Thirty devices were simultaneously produced in every fabrication run, and the cost of each ePAD, in terms of materials, was calculated as less than 0.2 €.

### Measurement Procedure

**Figure 2:**
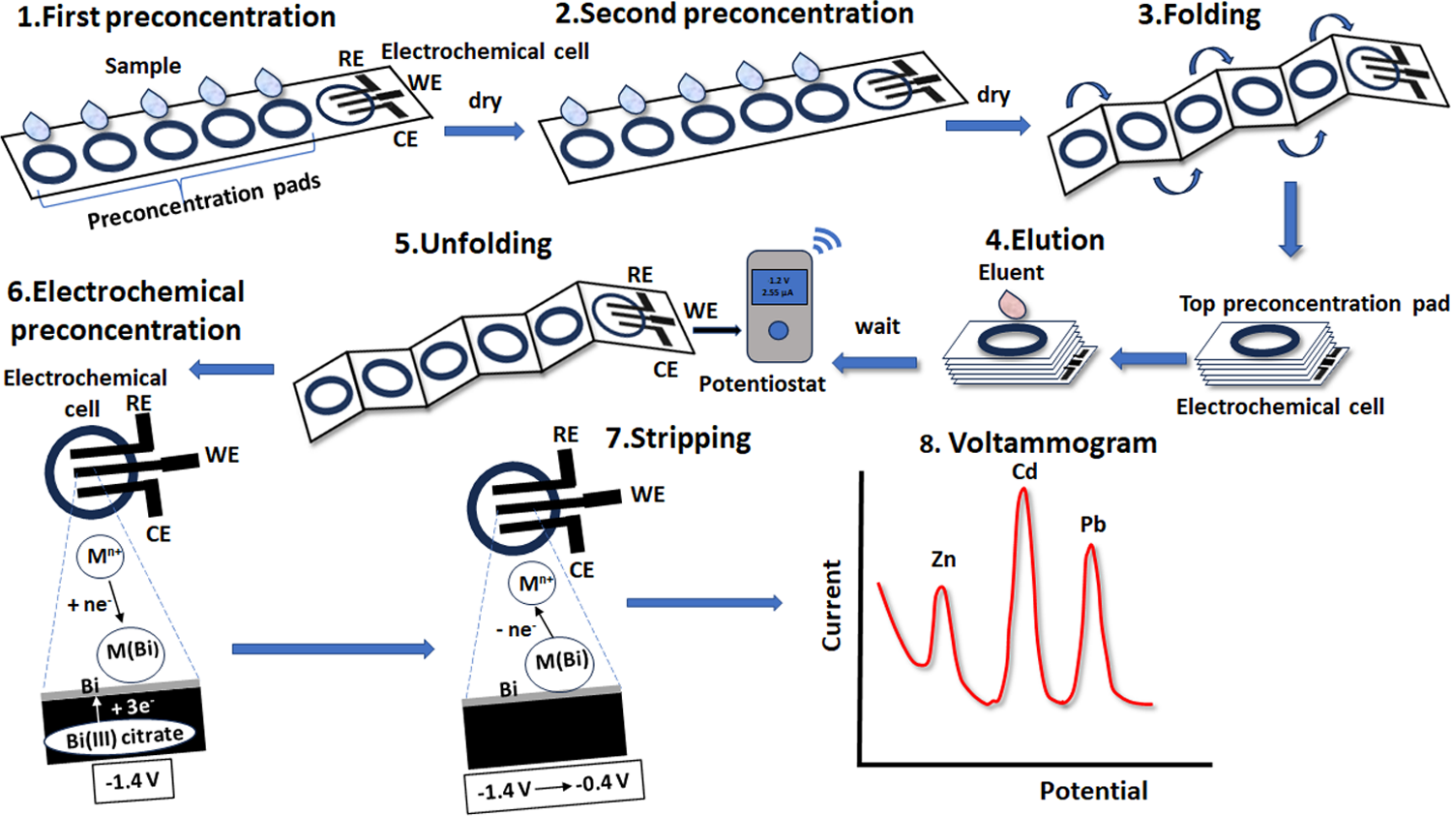
Experimental procedure for multiplexed heavy metal detection using the multifolding ePADs and dual passive preconcentration (M are the target metals and M(Bi) are the alloyed metals with bismuth; WE is the working electrode; RE is the reference electrode; and CE is the counter electrode).

The experimental procedure is illustrated in Figure 2 and is also depicted in the relevant video (ePAD.mp4, Supporting Information). 20 μL of sample was added in each of the five sample preconcentration pads of the multifolding ePAD; then, the solution was left to dry, and the process was repeated one more time. Then, the preconcentration layers were folded atop the electrochemical cell; 40 μL of 0.1 mol L^–1^ acetate buffer (pH 4.5) was added to the top zone, and then the buffer solution was allowed to flow vertically through the stacked preconcentration zones eluting the target analytes, and the final eluent was collected at the electrochemical cell at the bottom of the stack. After 30 s, the ePAD was unfolded, the 3 electrodes were connected to the portable potentiostat, and a preconcentration potential of −1.4 V vs the carbon quasi-reference electrode was applied to the working electrode. During the reductive potentiostatic preconcentration step, the bismuth citrate at the surface of the working electrode was reduced to metallic bismuth, and the target metal cations were simultaneously reduced to the respective metals and were immediately alloyed with bismuth. Finally, a voltammetric scan was applied from −1.4 to −0.4 V in the square wave mode (frequency 50 Hz, pulse height 25 mV, step 4 mV) in which the preconcentrated metals were oxidized and the voltammogram was recorded.

For comparative experiments with an electroplated bismuth-film working electrode, an ePAD with an unmodified carbon working electrode was used, and the electrochemical cell was preloaded with 50 μL of a 20 mg L^–1^ Bi(III) solution which was left to air-dry. The sample addition, preconcentration, and measuring steps were the same as described in the previous paragraph.

For comparative experiments without dual preconcentration, only the electrochemical cell of the folding ePAD was used. A 40 μL drop of sample was added to the electrochemical cell of the ePAD, the solution was left to dry, and 40 μL of 0.1 mol L^–1^ acetate buffer (pH 4.5) was added. The preconcentration and measurement steps were the same as described in the previous paragraph.

## Results and Discussion

### Characterization of the Working Electrode

**Figure 3:**
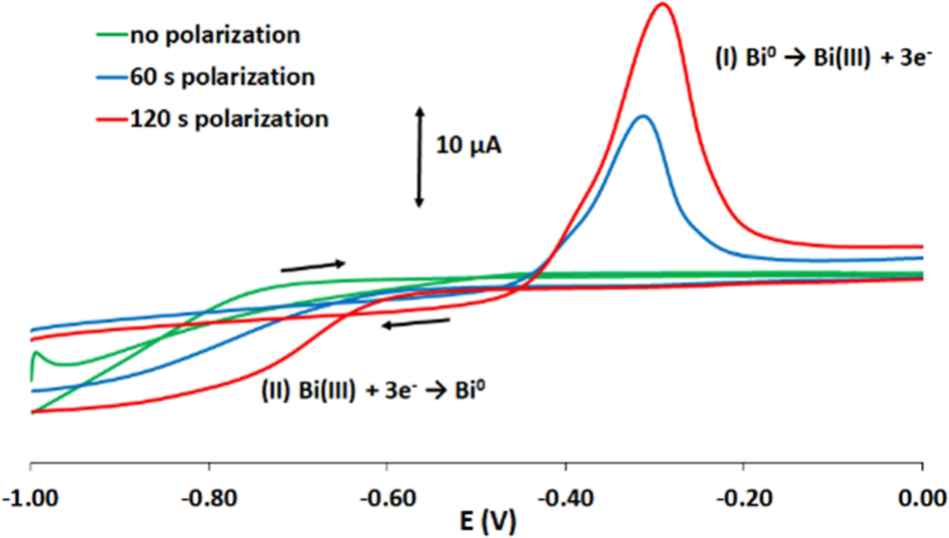
CVs of 0.1 mol L^–1^ acetate buffer (pH 4.5) in the electrochemical cell after different polarization times at −1.4 V.

Initially, the formation of metallic bismuth via the reduction of the bismuth citrate precursor was studied by placing 40 μL of 0.1 mol L^–1^ acetate buffer (pH 4.5) in the electrochemical cell of the folding ePAD and recording CVs in the range of −1.0 to 0.0 V. As illustrated in Figure 3 (green trace), when no reductive polarization step was applied to the working electrode, no bismuth oxidation and reduction signals were obtained in the CV scan. However, when a reductive polarization step at −1.4 V was applied to the working electrode for 60 s, a strong anodic peak (peak I) was obtained at −0.30 V in the forward anodic scan (Figure 3 (blue trace)); this peak was due to the oxidation of the metallic bismuth generated from the reduction of Bi(III) in bismuth citrate to metallic Bi. A wide cathodic sigmoidal-shaped signal (peak II) was also obtained in the reverse cathodic scan, and this was attributed to the partial reduction of Bi(III) back to metallic bismuth. The oxidation and reduction signal intensities increased when the reductive polarization step at −1.4 V was applied to the working electrode for 120 s reflecting the larger amount of metallic bismuth generated (Figure 3 (red trace)).

**Figure 4:**
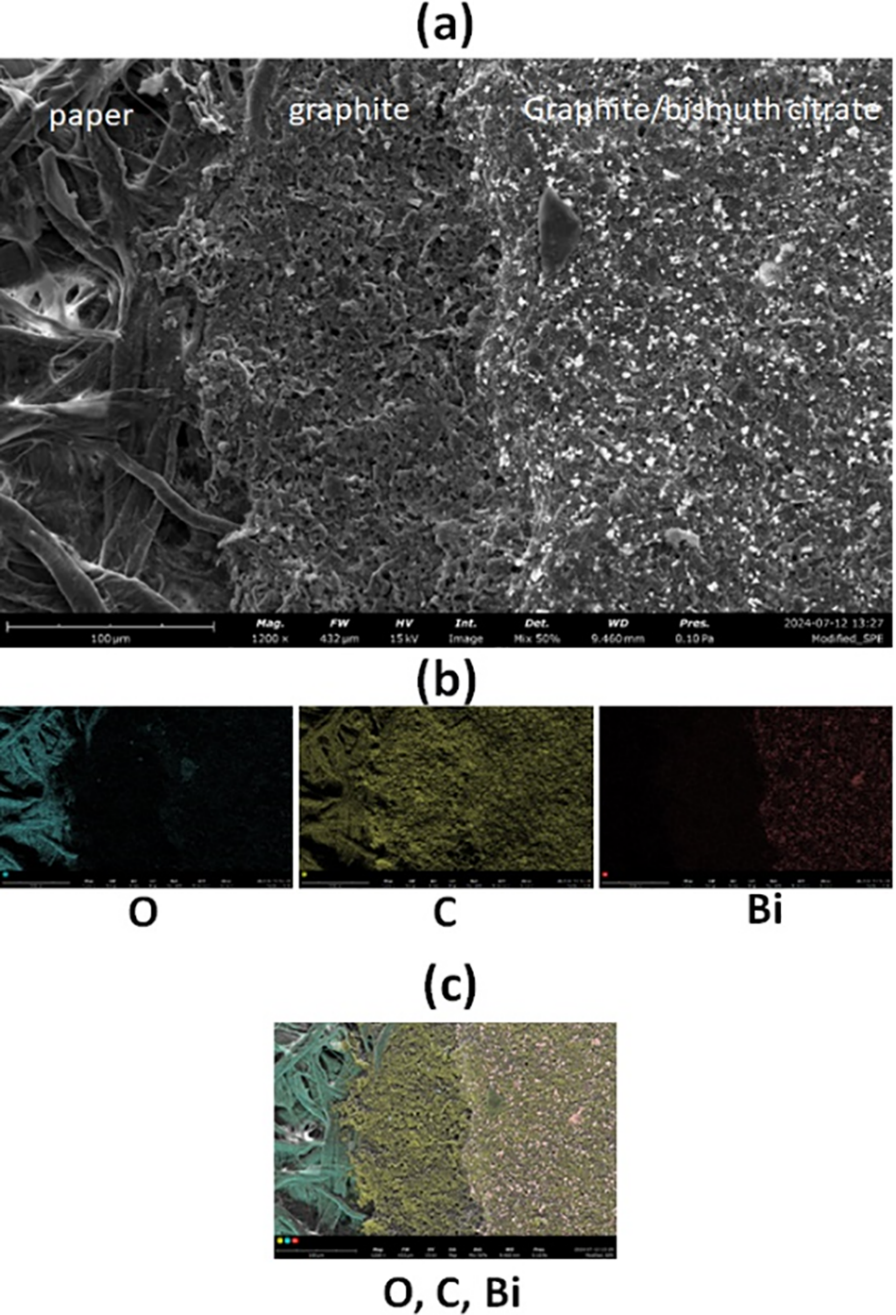
(a) SEM image at the interface between the paper substrate and the working electrode of the ePAD, (b) EDX mappings for O, C and Bi at the different regions of the interface, and (c) overlaid EDX mappings for O, C and Bi at the interface.

The morphology of the working electrode surface was also studied by SEM and EDX after placing 40 μL of 0.1 mol L^–1^ acetate buffer (pH 4.5) in the electrochemical cell of the folding ePAD and undergoing potentiostatic polarization at −1.4 V for 120 s. As illustrated in Figure 4a, the SEM image clearly differentiates between the paper substrate, the first layer of the screen-printed electrode (graphite), and the second layer of the screen-printed electrode (graphite loaded with bismuth citrate). As shown in the EDX mapping (Figure 4b), oxygen is present only in the cellulose paper substrate and carbon is present in both the paper substrate and the screen-printed electrode, while bismuth nanodomains are uniformly distributed only at the layer of the screen-printed electrode loaded with bismuth citrate. The elemental compositions of the three distinct regions of the electrochemical cell are also illustrated in the overlaid EDX mapping in Figure 4c.

### Study of the ePAD Operational Parameters

**Figure 5:**
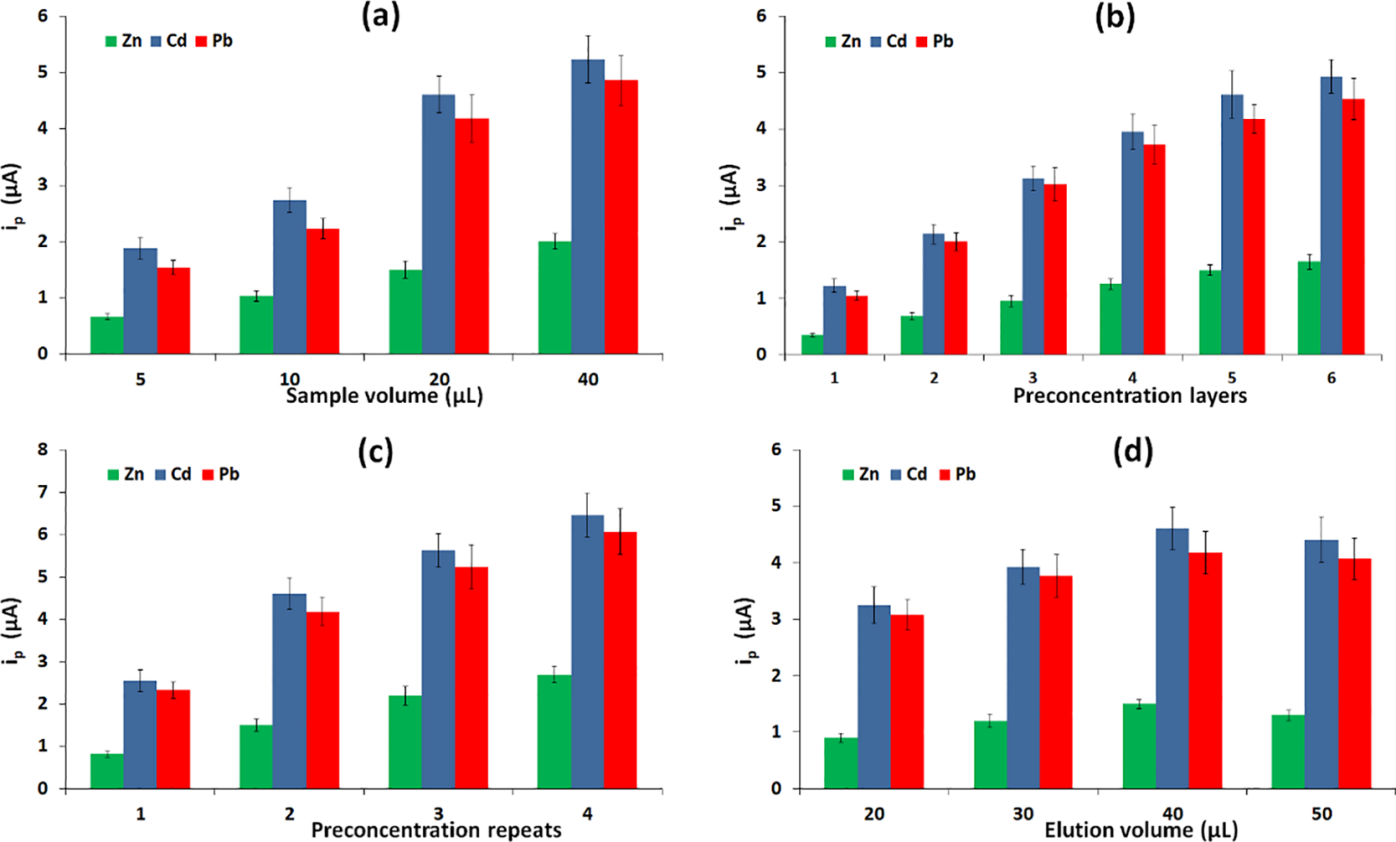
Effect of the (a) sample volume, (b) number of preconcentration layers, (c) number of sample repeats, and (d) elution volume on the peak current in a solution containing 15 μg L^–1^ of the target metals.

Different parameters pertaining to the geometry, the spatial configuration, and the physical operation of the multifolding ePAD were studied by analyzing a mixed standard solution containing the target metal cations (Cd(II), Pb(II), and Zn(II)) using ASV. Initially, the effect of the sample volume was evaluated in the range of 5–40 μL using a device composed of 5 preconcentration layers and 2 sample repeats (Figure 5a). The smaller sample volumes (5 and 10 μL) produced lower signals as the total amount of passively preconcentrated target metals was lower. Higher sample volumes produced larger signals, but the drying time was significantly increased and a volume of 20 μL was finally selected. Another important parameter was the number of preconcentration layers integrated into the device, which was varied from 2 to 6 (Figure 5b). When fewer preconcentration layers were used, the detection sensitivity was lower due to the lower total amount of heavy metals deposited on the device and ultimately eluted to the electrochemical cell. As the number of preconcentration layers increased, the sensitivity gradually increased along with the total amount of the target species preconcentrated on the device and the response started to level off for more than 5 preconcentration layers. This behavior is attributed to the fact that, when many preconcentration layers are used in tandem, the analytes eluted from the upper zones have increased chances and sufficient time to readsorb at the lower downstream zones, making the elution process less effective. Therefore, in this work, 5 preconcentration layers were finally selected. Next, the number of sample preconcentration repeats was studied (Figure 5c). For this task, the sample repeats were varied from 1 to 4 on the 5 sample zones, and the results suggested an increase in the response with an increasing number of repeats since more of the target analytes were deposited on the ePAD. However, the signal increase at more repeats was not linear, presumably because only part of the preconcentrated metals was ultimately eluted to the electrochemical cell. In addition, increasing the number of the sample preconcentration repeats resulted in extended analysis time since the assay time was directly proportional to the number of repeats due to the requirement for sample drying between successive repeats; in this work, 2 sample preconcentration repeats were used as a compromise between adequate sensitivity and short assay time. Next, the volume of the buffer solution used for the elution of the analytes was evaluated in the range of 10–50 μL (Figure 5d). For elution volumes lower than 10 μL, the volume of the eluent was not sufficient to penetrate all the preconcentration zones and reach the electrochemical cell. For elution volumes of 20–40 μL, the signal increased with increasing volume, but at volumes >40 μL, the signal started to decrease. This effect can be accounted for by the assumption that two opposing effects were at play, namely elution efficiency and dilution of the eluate. As the elution volume increased in the range of 10–40 μL, the elution efficiency was improved and compensated for the higher dilution of the target metals in the eluate, thus causing a net signal increase. However, at volumes >40 μL, the dilution of the analytes in the final eluate started to become a more significant factor, therefore causing a net signal decrease. An elution buffer volume of 40 μL was used in the following experiments.

As demonstrated in the voltammograms in Figure S1a (Supporting Information), the use of the multifolding ePAD with the dual preconcentration step afforded an approximate 6-fold sensitivity enhancement with respect to the case of an ePAD without prior preconcentration step. A comparison between working electrodes bulk-modified with bismuth citrate and electroplated with bismuth suggested that the former produced higher signal amplification (especially for Pb(II) and Zn(II)) while also requiring a simpler analytical protocol (Figure S1b, Supporting Information).

### Study of the Chemical and Instrumental Parameters

**Figure 6:**
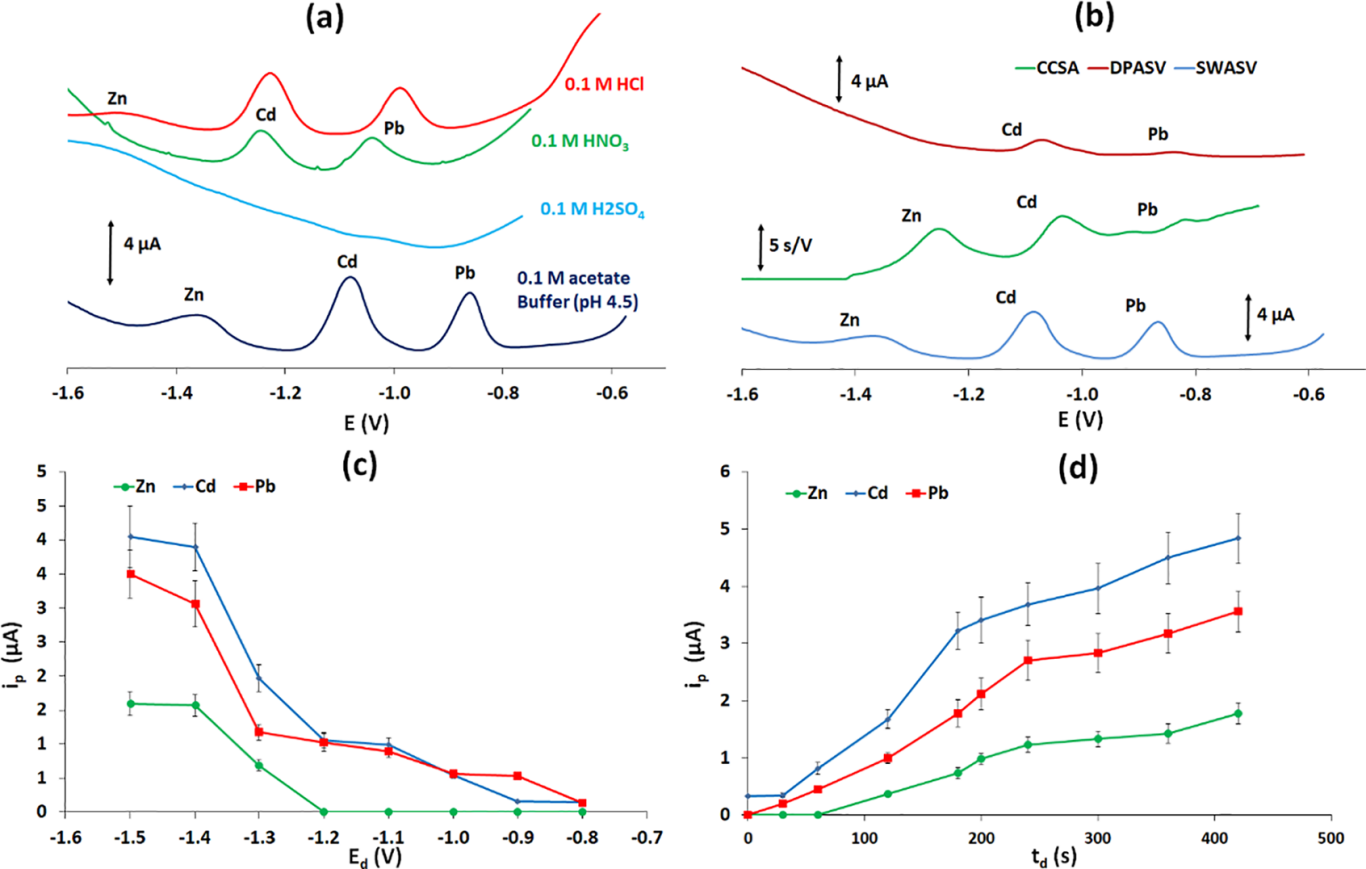
Effect of the (a) elution electrolyte, (b) stripping technique, (c) deposition potential, and (d) deposition time on the peak current in a mixed solution containing 15 μg L^–1^ of the target metals.

Different detection parameters were studied including the elution buffer, the stripping technique, the deposition time, and the deposition potential. First, the type of elution buffer was studied using 0.1 mol L^–1^ H_2_SO_4_, HNO_3_, HCl, and acetate buffer (pH 4.5) for eluting the target metals to the electrochemical cell (Figure 6a). It must be noted that the elution buffer determines both the elution efficiency and the accumulation and stripping conditions during the detection step. As illustrated in Figure 6a, the best response for multiplexed detection was obtained using 0.1 mol L^–1^ acetate buffer (pH 4.5) as the eluent. Then, the stripping technique was selected by comparing differential-pulse anodic stripping voltammetry (DPASV), square wave anodic stripping voltammetry (SWASV), and constant-current stripping analysis (CCSA) (Figure 6b) with the square wave waveform producing the highest sensitivity. The effect of the preconcentration potential was studied in the range of −0.80 to −1.50 V (Figure 6c). The effect of the preconcentration potential was two-fold since it determined both the extent of the reduction of bismuth citrate within the precursor-modified electrode and the reductive preconcentration of the target metals. The response for the three metals at potentials more positive than −1.0 V was minimal as neither bismuth citrate nor the target cations were reduced to any considerable extent. The signals increased as the deposition potential became more negative, approaching a plateau when the mass-transfer-limited region of each metal was eventually reached. The deposition time was evaluated in the range of 0–420 s (Figure 6d); the signals increased sharply with increasing deposition time up to 240 s and then started to level off at higher deposition times due to depletion of the cations around the vicinity of the working electrode. The judicious choice of the deposition time and potential allows one to tailor the magnitude of the relative individual responses of the target metals.

### Analytical Features and Applications

**Figure 7:**
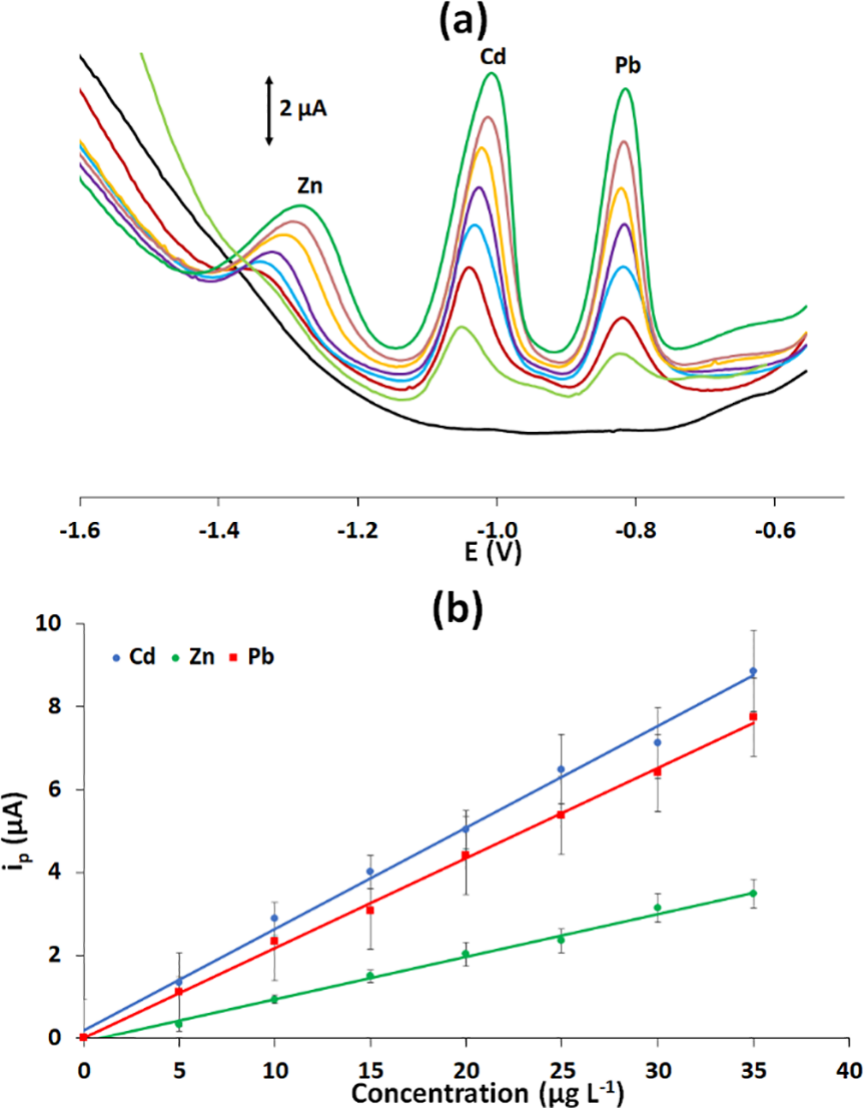
(a) Voltammograms of the target metals in the concentration range of 0–35 μg L^–1^. (b) Respective calibration plots (each point is the average of the three different measurements).

The folding ePADs were tested for multiplexed determination of Cd(II), Pb(II), and Zn(II) in the concentration range of 5–35 μg L^–1^, and respective voltammograms for a single series of measurements are illustrated in Figure 7a. The calibration plots for the 3 target metals (calculated as an average of three different calibration series) are illustrated in Figure 7b. The LODs (calculated as three times the standard deviation of intercept divided by the slope of the calibration plot) were 0.9 μg L^–1^ for Cd(II), 0.6 μg L^–1^ for Pb(II), and 2.7 μg L^–1^ for Zn(II). No fully integrated PADs have been reported in the literature for multielement determinations by stripping analysis, but these LODs are significantly lower than those for duplex heavy metal detection (i.e., Pb(II) and Cd(II)) using integrated ePADs,^22,24,26^ a corollary of high preconcentration efficiency of the dual preconcentration methodology (Table S1, Supporting Information). Since each ePAD was used for a single measurement, the repeatability was determined by the variability both in the ePAD fabrication process and in the analytical protocol, and the coefficients of variation ranged from 9 to 16%.

In addition, the effect of some possible interferences (Mn(II), Ni(II), Mg(II), Fe(III), Sb(III), Sn(II), and Cu(II)) was studied. The signals of 20 μg L^–1^ of Pb(II), Cd(II), and Zn(II) were not statistically affected by a 10-fold mass concentration excess of Mn(II), Ni(II), Mg(II), and Fe(III). The effect of Sb(III), Sn(II), and Cu(II)) is illustrated in Figure S2 (Supporting Information). Since Cu(II) is expected to exist at higher concentrations with respect to Pb(II) and Cd(II) in many samples, its interference was deemed the most serious. Cu(II) has been known to affect the Cd and Pb peaks due to the formation of intermetallic compounds and the deposition of the target metals on electroplated Cu and is effectively alleviated by the addition of ferrocyanide in the sample.^54^

**Figure 8:**
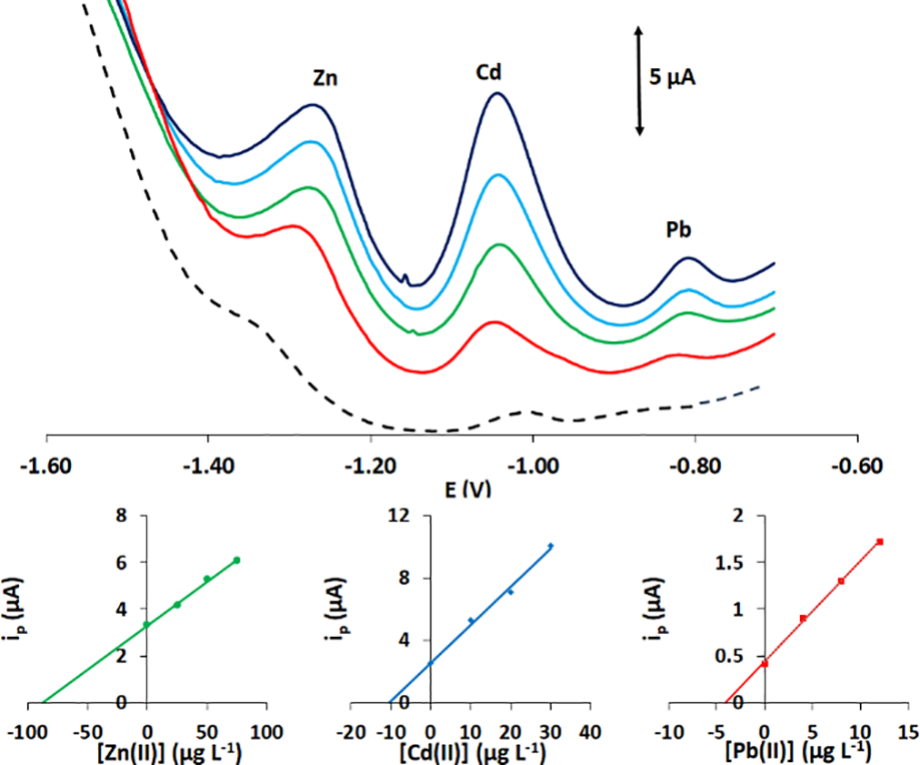
Voltammograms and standard addition plots for the determination of Zn, Cd, and Pb in a phosphate fertilizer sample using the method of standard additions. Lower dotted trace is the voltammogram without dual preconcentration; red trace is the voltammogram of the sample; green, light blue, and dark blue traces are the voltammograms of the sample fortified with the target metals.

The multifolding ePADs were applied to the determination of the target metals in a phosphate fertilizer sample (which was treated and analyzed as described in the Supporting Information) using the method of standard additions. Since the sample contained a large excess of Zn, deposition was performed at a more positive potential of −1.3 V in order to suppress the Zn deposition and to avoid overlap of the prominent Zn peak with the Cd peak. Respective voltammograms of the sample and the fortified sample with the target trace metals are illustrated in Figure 8 (colored traces); the voltammogram without dual preconcentration is shown as the dotted trace demonstrating the effectiveness of the preconcentration procedure. The standard additions plot suggests that the sample contains 88 ± 9 μg g^–1^ of Zn, 10.6 ± 1.3 μg g^–1^ of Cd, and 4.1 ± 0.6 μg g^–1^ of Pb, values that are within the expected range for this type of sample.^55^ The sample was analyzed with atomic absorption spectroscopy (Supporting Information) with calculated values of 94 ± 8 μg g^–1^ for Zn, 11.5 ± 1.0 μg g^–1^ for Cd, and 4.7 ± 0.5 μg g^–1^ for Pb with a relative bias <10% for all three target metals.

The ePADs were also applied to the determination of the target metals in a honey sample (which was treated and analyzed as described in the Supporting Information) using the method of standard additions. Analysis of the sample indicated that the content in the target metals was below the LOD of the method. The accuracy was ascertained by spiking the sample with 0.1 mg kg^–1^ of the target metals (0.1 mg kg^–1^ is the maximum limit set by the EU for Pb,^56^ while there are no legislative limits for Zn and Cd) and calculating the recovery. Respective voltammograms of the spiked sample before and after the additions are illustrated in Figure S3 (Supporting Information); the % recoveries were calculated as 105% for Zn, 103% for Cd, and 97% for Pb.

## Supporting Information Available

Preparation of the honey sample; preparation of the phosphate fertilizer sample; Figure S1: comparison of the voltammograms of a solution containing 20 μg L^–1^ of the target metals with dual preconcentration and without preconcentration, using a working electrode modified with bismuth citrate and a working electrode electroplated with bismuth; Table S1: comparison of paper-based devices for duplex heavy metal determination; Figure S2: voltammograms of a solution containing 15 μg L^–1^ of Ζn(ΙΙ), Cd(II), and Pb(II) in the presence of 60 μg L^–1^ of various potentially interfering cations; Figure S3: voltammograms and standard addition plots for the determination of Zn, Cd, and Pb in a spiked honey sample with 0.1 mg kg^–1^ of Zn(II), Cd(II), and Pb(II) using the method of standard additions (PDF)
Short video of the experimental procedure (MP4)

## Abbreviations

ePAD: electrochemical paper-based device
ASV: anodic stripping voltammetry
LOD: limit of detection
WE: working electrode
RE: reference electrode
CE: counter electrode
SEM: scanning electron microscopy
EDX: energy-dispersive X-ray spectroscopy
