# Supplementary material for: Multifolding Vertical-Flow Electrochemical Paper-Based Devices with Tunable Dual Preconcentration for Enhanced Multiplexed Assays of Heavy Metals
Source: Anal Chem. 2025 Jan 9;97(2):1457–64. doi: 10.1021/acs.analchem.4c06982 (PMC11755402; doi:10.1021/acs.analchem.4c06982)
Supplement: Supplementary file 1 — ac4c06982_si_001.pdf [file ac4c06982_si_001.pdf]

Supporting Information

Multifolding vertical-flow electrochemical paper-based devices with tunable dual preconcentration for enhanced multiplexed assay of heavy metals

Dionysios Soulis[1], Electra Mermiga[1], Varvara Pagkali[1], Maria Trachioti[2], Christos Kokkinos[1], Mamas Prodromidis[2], Anastasios Economou[1]*

[1] *Laboratory of Analytical Chemistry, Department of Chemistry, University of Athens, Athens 157 71, Greece.*

[2]*Laboratory of Analytical Chemistry, Department of Chemistry, University of Ioannina, Ioannina 45110, Greece.*

* Email: aeconomo@chem.uoa.gr

Table of content

	Page
Preparation of the phosphate fertilizer samples	S2
Preparation of the honey samples	S2
Figure S1	S3
Table S1	S4
Figure S2	S5
Figure S3	S6
Short video of the experimental procedure	S7

Preparation of the phosphate fertilizer samples

0.1 g of an accurately weighed phosphate fertilizer sample was digested almost to dryness in 5 mL HNO_3 (70 %) over a hotplate and the residue was dissolved and brought a final volume of 100 mL with the addition of 0.1 mol L^{-1} acetate buffer (pH 4.5) and 100 μL of a 1.0×10^{-2} mol L^{-1} $K_4[Fe(CN)_6]$ solution was added (to achieve a final 1.0×10^{-5} mol L^{-1} concentration of $K_4[Fe(CN)_6]$). Three fortified fertilizer samples were prepared by further adding, respectively: 25 μg L^{-1} of Zn(II), 10 μg L^{-1} of Cd(II), 5 μg L^{-1} of Pb(II); 50 μg L^{-1} of Zn (II), 20 μg L^{-1} of Cd(II) and 10 μg L^{-1} of Pb(II); 75 μg L^{-1} of Zn(II), 30 μg L^{-1} of Cd(II) and 15 μg L^{-1} of Pb(II), in the treated fertilizer sample. The samples were also analyzed with a Perkin Elmer 5100 AAS spectrometer (CT, USA) equipped with a Perkin Elmer 5100 ZL furnace module.

Preparation of the honey samples

The honey sample was purchased from a local supermarket. 0.4 g of the honey sample was accurately weighed and was digested almost to dryness in 5 mL HCl (30%) and 1 mL H_2O_2 (30%) over a hotplate. The solution was brought to a final volume of 10 mL with addition of 0.1 mol L^{-1} acetate buffer (pH 4.5) and 10 μL of a 1.0×10^{-2} mol L^{-1} $K_4[Fe(CN)_6]$ solution was added (to achieve a final 1.0×10^{-5} mol L^{-1} concentration of $K_4[Fe(CN)_6]$). A spiked honey sample was prepared by spiking 0.4 g of accurately weighed honey sample with 10 μL of the mixed solution containing 10 mg L^{-1} Pb(II), Cd(II) and Zn(II) and was treated as above. The final concentration in the treated sample was 25 μg L^{-1} for the three target cations. Two fortified spiked honey samples were prepared by further adding 15 and 30 μg L^{-1} of the three target metals in a treated spiked honey sample.

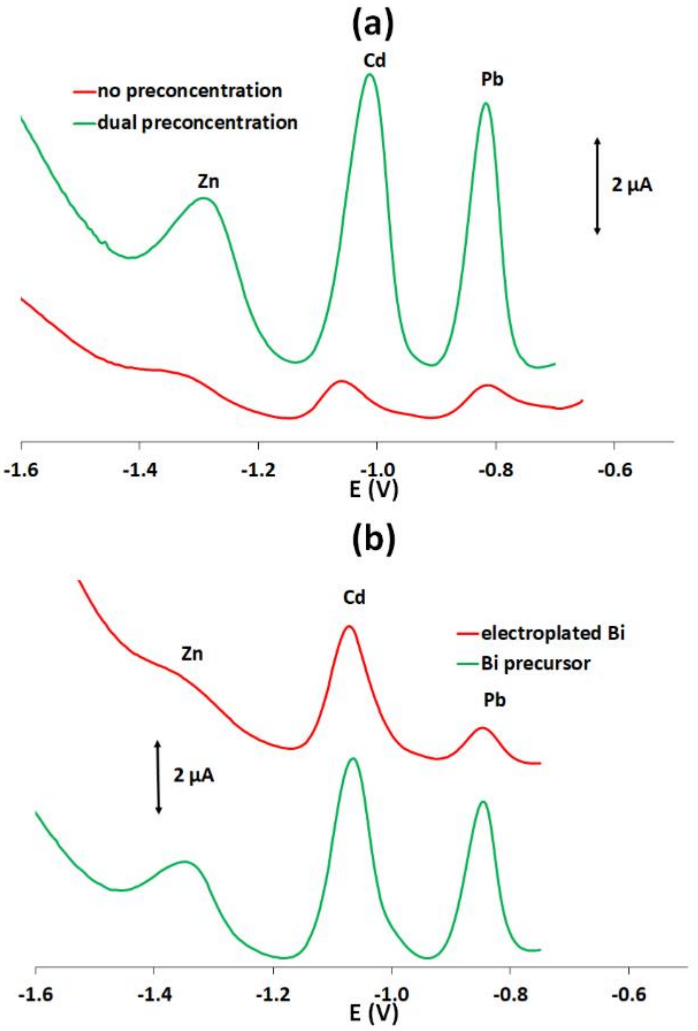

Figure S1. Comparison of the voltammograms of a solution containing 20 µg L^{-1} of the target metals (a) with dual preconcentration and without preconcentration, (b) using a working electrode modified with bismuth citrate and a working electrode electroplated with bismuth.

Table S1. Paper-based devices for duplex heavy metals determination (references from the main text).

Target metal(s)	Limits of detection ($\mu g\ L^{-1}$)	Type of device	Modifier	Ref.
Pb, Cd (childrens' shoes)	1(Pb(II)), 5(Cd(II)	Paper disk on ITO-carbon cement WE (non-integrated)	-	[15]
Pb, Cd (water)	100(Pb(II)), 400(Cd(II)	Paper disk on screen-printed sensor (non-integrated)	Hg, Bi	[19]
Pb, Cd (water)	1(Pb(II)), 2.4(Cd(II))	Paper disk on screen-printed sensor (non-integrated)	Bi	[20]
Pb, Cd (air)	1(Pb(II)), 1(Cd(II))	Paper fluidic on screen-printed sensor (non-integrated)	Bi	[21]
Pb, Cd (fish food, water)	4.5(Pb(II)), 3.1(Cd(II))	Pen-plotted fluidic and sensor (integrated)	Bi	[22]
Cd, Pb (water)	4.2(Pb(II)), 2.4(Cd(II))	Paper fluidic on screen-printed sensor (integrated)	Bi	[24]
Cd, Pb (mud, seawater)	7(Pb(II)), 11(Cd(II))	Paper fludic on screen-printed sensor (integrated)	-	[26]
Pb, Cd (aerosols)	0.5(Pb(II)), 0.5(Cd(II))	Paper fluidic on screen-printed sensor (non-integrated)	Bi	[28]
Pb(II), Cd(II) (water)	1(Pb(II)), 25(Cd(II))	Paper fluidic on stencil-printed sensor (non-integrated)	Bi	[34]

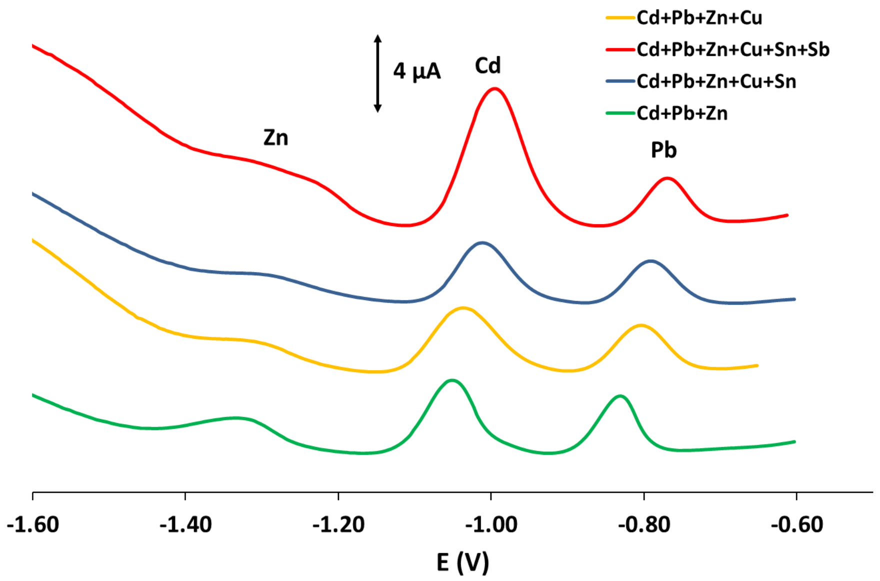

Figure S2. Voltammograms of a solution containing 15 μg L^{-1} of Zn(II), Cd(II) and Pb(II) in the presence of 60 μg L^{-1} of various potentially interfering cations.

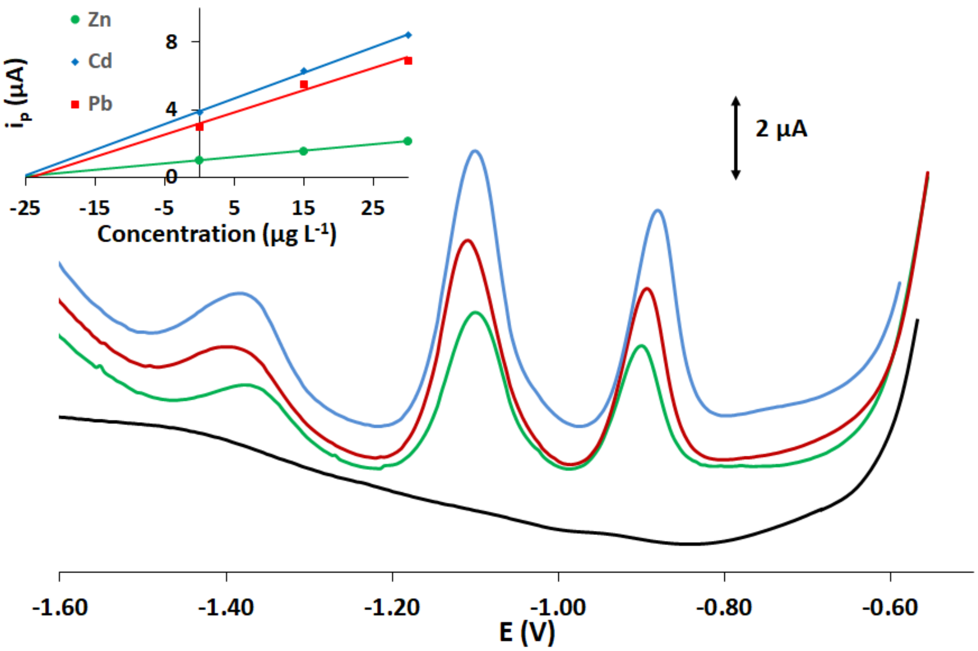

Figure S3. Voltammograms and standard addition plots for the determination of Zn, Cd and Pb in a honey sample spiked with 0.1 mg Kg^{-1} of Zn(II), Cd(II) and Pb(II) using the method of standard additions. The lowest black trace is the voltammogram of the unspiked sample; the green trace is the voltammogram of the spiked sample; the red and blue traces are the voltammograms of the fortified samples.

Short video of the experimental procedure

The supplementary video ePAD.mp4 briefly describes in a video format the four main steps of the experimental procedure for the multiplexed assay of heavy metals using the folding ePADs: passive preconcentration (x2); folding; elution, and; unfolding and ASV measurement.
